# Relationship of Hypertensive Retinopathy with Mean Platelet Volume Among Hypertensive Patients

**DOI:** 10.7759/cureus.422

**Published:** 2015-12-21

**Authors:** Adnan Bashir Bhatti, Farhan Ali, Siddique Akbar Satti

**Affiliations:** 1 Department of Medicine, Capital Development Authority Hospital, Islamabad, Pakistan

**Keywords:** mean platelet volume, platelet activation, essential hypertension, retinopathy, hypertensive retinopathy

## Abstract

Background The pathophysiological mechanism of hypertensive retinopathy (HR) is not fully understood, although it is thought that increased platelet activation may have a pivotal role. This study was designed to investigate this possibility by determining the frequency and magnitude of mean platelet volume (MPV), a marker of platelet activation, in HR.

Methods One hundred and thirty-eight patients, aged 31 to 80 years, who had essential hypertension with HR were enrolled in the study. The patients who presented at the emergency and outpatient medical department of the Capital Development Authority (CDA) Hospital, Islamabad, Pakistan, from March 2013 to February 2014 were selected for this study. All patients were analyzed for grading of HR according to the Keith-Wagener-Barker (KWB) classification criteria. A direct ophthalmoscopic examination was performed in all the subjects, together with an assessment of MPV.

Results The mean age of the patients was 54.1 ± 11.7 years. Normal MPV was found in 97 (70.2%) patients, 53 males and 44 females. Elevated levels of MPV were found in 41 (29.7%)  patients, 19 males and 22 females. In patients with Grade 1 HR, the MPV was 10.6 ± 0.6 femtoliters (fl). In Grades 2, 3, and 4 HR, the values of MPV were 11.1± 0.5 fl, 11.6 ± 0.3 fl, and 12.2 ± 0.6 fl, respectively, which were greater than the normal range (> 10 fl) of MPV values. In addition, the frequency of elevated MPV significantly (P < 0.001) and linearly (r = 0.998) increased with increasing HR grade.

Conclusion It is concluded that the frequency of elevated MPV is increased in patients with HR and strongly correlates to grade. In addition, the magnitude of the elevated MPV increased with the severity of the retinopathy. Thus, abnormally elevated MPV may be an etiological factor for HR in hypertensive patients.

## Introduction

Hypertension is one among the most common cardiovascular diseases reported in Pakistan. The National Health Survey of Pakistan (NHSP) estimated the prevalence of hypertension among adults over 18 years as 18% and in those over 45 years as 33% [1]. Poorly managed hypertension is often associated with end organ damage, including hypertensive retinopathy (HR) [2-3]. Hypertensive retinopathy is a disease characterized by a range of retinal vascular signs in patients with hypertension [4]. HR is classified into four grades on the basis of clinical descriptions. Grade 1: Mild generalized retinal arteriolar narrowing; Grade 2: Definite focal narrowing and arteriovenous nipping; Grade 3: Signs of Grade 2 retinopathy, plus retinal hemorrhages, exudates, and cotton-wool spots, and Grade 4: Severe Grade 3 retinopathy, plus papilledema [5]. The prevalence of HR among hypertensive patients is quite high, seen in both sexes, and almost in all age groups [6-7]. It increases with duration of the disease, attaining a maximum severity after 10 years [7]. Generalized retinal arteriolar narrowing and arteriovenous nicking are known to be related to chronically high blood pressure [8]. Hypertension alone does not fully account for the development or extent of retinopathy; other pathogenic factors, such as elevated platelet activation, may also be involved [3]. The purpose of this study was to further investigate this possibility by measuring platelet volume in patients diagnosed with HR [6]. Platelet volume is a marker of the platelet function and activation, and it is determined as mean platelet volume (MPV) [9].

## Materials and methods

This study was comprised of 138 patients (age range: 31 to 80 years, mean ± SD: 54.1 ± 11.7 years) presenting in the emergency and outpatient medical department of Capital Development Authority (CDA) Hospital, Islamabad, Pakistan, a tertiary care hospital. Informed patient consent was obtained from all patients. The Human Ethics Committee of the Capital Development Authority (CDA) Hospital, Islamabad, Pakistan approved this study. Data collection was started from March 2013 to February 2014. The patients were selected via non-probability convenient sampling. All were hypertensive with HR. Exclusion criteria were carefully screened in order to avoid the interference of extrinsic factors that may influence the MPV values. Patients with major illnesses and health conditions/habits like diabetes mellitus, dyslipidemia, obesity, smoking, and on drug therapy that might interfere with MPV (e.g., warfarin) and those who underwent recent surgery were excluded from the study.

The same clinicians examined and collected data on all patients in order to prevent bias. Arterial blood pressure was recorded using a mercury sphygmomanometer after the subject had taken a five-minute rest. For each subject, an average of two readings were obtained. Hypertension was defined as systolic blood pressure ≥ 140 mmHg and diastolic blood pressure ≥ 90 mmHg. Blood samples were drawn after a fasting period of 12 hours and collected in citrate (1:4 v/v) in order to reduce platelet swelling induced by ethylenediaminetetraacetic acid (EDTA). The test was performed in the Sysmex Autoanalyzer (Sysmex Corp., Kobe, Japan) within 30 minutes after the collection of a sample to reduce the variations in results due to sample aging. The direct ophthalmoscopic examinations were done after dilatation of pupils with Mydriacyl (tropicamide) 1% eye drops for the evaluation of HR. 

### Statistical evaluation

The Statistical Package for Social Sciences (SPSS, version 14.0) was used to enter and analyze the data in the form of tables and graphs. Mean, frequencies, percentages, and p-values were calculated.

## Results

Of the 138 patients with varying degrees of HR, 72 (52.2%) were male and 66 (47.8%) were female. Elevated MPV > 10 femtoliter (fl) was observed in 41 cases (29.7%). The sex distribution of the latter was 19 males (26.3%), 22 females (33.3%) (Table 1).

**Table 1:** Percentage of normal and elevated mean platelet volume (MPV) among the sexes in patients with hypertensive retinopathy (HR) ^a^Male vs. Female distribution, not significant (P > 0.05 by Chi-squared)

Sex	Number with Normal MPV	Number with Elevated MPV	Percentage with Elevated MPV^a^
M	53	19	26.3%
F	44	22	33.3%
Total	97	41	29.7%

Tables 2-3 summarize the relationship between the percentage of patients with elevated MPV with age and grade of HR, respectively. Increased frequency of MPV was observed with increasing age of the subjects (Table 2) with an r-value of 0.847, although this was not statistically significant (P = 0.07) at the 5% level. It is observed that the percentage of increased MPV cases was the highest for the age group > 70 years. 

**Table 2:** Percentage of mean platelet volume (MPV) in different age groups. ^a ^r = 0.847, P = 0.07

Age Group	Number with Normal MPV	Number with Increased MPV	Percentage in Group with Increased MPV^a^
31 - 40	15	5	25.00%
41 - 50	23	11	32.35%
51 - 60	26	6	18.75%
61 - 70	29	15	34.10%
71 - 80	4	4	50.00%
Total	97	41	25.90%​

**Table 3:** Percentage of mean platelet volume (MPV) according to grade of hypertensive retinopathy (HR). ^a^ r = 0.998, P < 0.001

HR Grade	Number with Normal MPV	Number with Elevated MPV	Percentage in Group with Elevated MPV^a^
1	49	9	15.50%
2	41	21	33.90%
3	6	8	57.10%
4	1	3	75.00%
Total	97	41	25.90%

**Figure 1:**
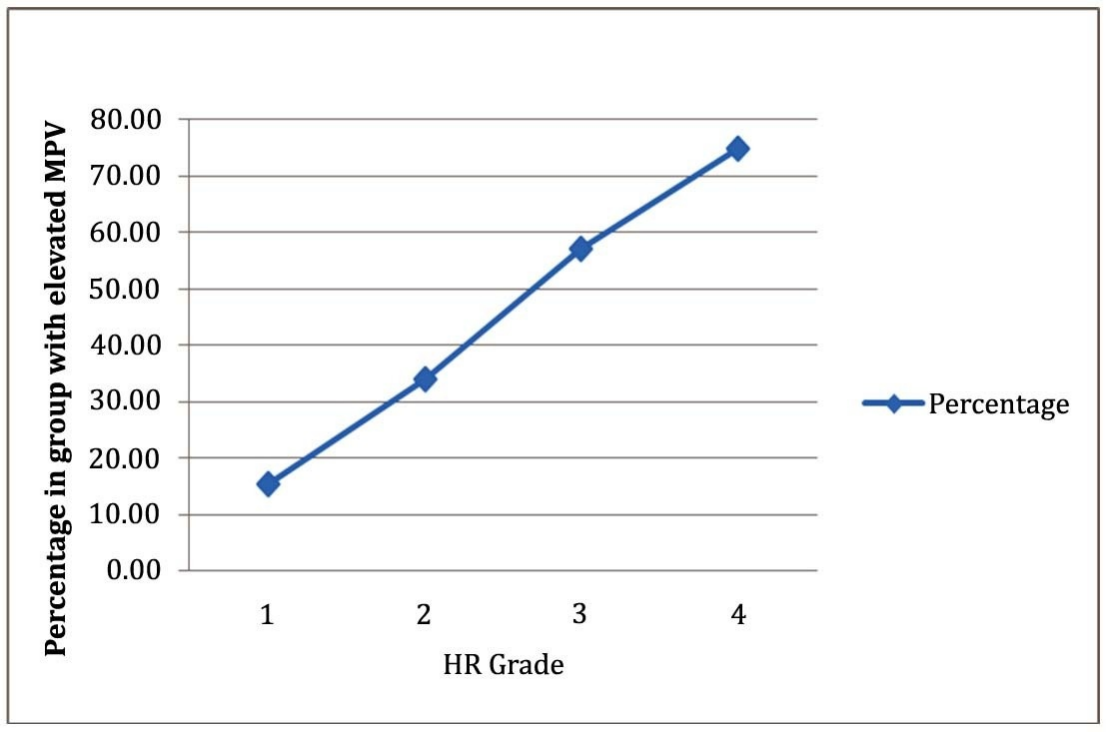
Linear relationship between frequency of elevated MPV and increasing grade of HR* * R = 0.998, P < 0.001

Figure 1 depicts the linear relationship between the grade (severity) of HR and frequency of elevated MPV, derived from the data in Table 3. There is a strong, positive, and statistically significant correlation (Pearson's product-moment correlation coefficient, r = 0.998, P < 0.001) between an increase in HR grade and an elevated MPV.  

In addition, in patients of Grade 1 HR, the MPV was 10.6 ± 0.6 fl. In Grades 2, 3, and 4 HR, the values of MPV were 11.1± 0.5 fl, 11.6 ± 0.3 fl, and 12.2 ± 0.6 fl, respectively (P < 0.05 by Analysis of Variance (ANOVA)), indicating a positive near linear correlation (r = 0.53).

## Discussion

Untreated or uncontrolled hypertension is often associated with serious vascular problems, such as stroke, cardiac abnormalities, and retinopathy [2-3, 6-7, 10-11]. It has been previously reported that the endothelial function of the retinal vasculature is impaired in early essential hypertension [12]. HR is a condition in which the retinal vessels respond to an increase in blood pressure by generalized arteriolar constriction. This will in turn lead to more pathological conditions, like cotton-wool spots, arteriolar necrosis, hemorrhage, retinal edema, and disc edema [13]. The elevated blood pressure alone does not appear to account for these complications. There is growing evidence that increased platelet activation, a common finding in hypertension, may contribute significantly to these complications [6, 10-11, 14]. Thus, it is important to assess the role of platelet activation in the disease process as well as to evaluate its use as a predictor for complications, including HR. Although there are a number of biochemical indicators of platelet activation, such as levels of beta-thromboglobulin and soluble P-selectin, most of these involve somewhat complicated and expensive laboratory assessment [15-16]. Platelets normally circulate in a quiescent disc-shaped state, and when activated, they transform into a sphere shape, resulting in an increase in size [17]. Consequently, MPV is an indicator of platelet size and has been known to be a marker of platelet activity [9]. Large platelets are more reactive and, therefore, will synthesize more thromboxane A_2_ and will aggregate more easily [18-19]. Patients with large sphere-shaped platelets can easily be detected through hematological analysis and, thus, could possibly benefit from preventive treatment. Thus, MPV is an easy and cost-effective test that can be used extensively in developing countries like Pakistan for forecasting the vascular dysfunctions [20].

For these reasons, the present study was conducted to help further evaluate the frequency and magnitude of increased MPV in patients with HR. The results demonstrated a statistically significant and linear relationship (r = 0.998, P < 0.001) between the grade of retinopathy in hypertensive subjects and frequency of elevated MPV. These findings concur with the result of a previous study [6]. Thus, it is a highly relevant finding that MPV in patients with hypertension is significantly higher than in normotensive control subjects [21]. Indeed, the latter and other studies also showed that within hypertensive groups, those with evidence of target organ damage, including HR, possessed significantly larger platelets, elevated platelet counts, altered platelet distribution width, and greater platelet large cell ratio than those without target organ damage [5-22]. The underlying relationship between hypertension and HR is clear, as retinopathy is often a product of arteriolar damage from raised blood pressure [5]. Increased MPV has additionally been shown to be associated with white coat syndrome and essential hypertensive subjects, indicating it might be a contributing factor to increased risk of developing microvascular complications [21]. This earlier study showed that MPV was significantly higher in essential hypertensive's patients than in normotensive control subjects. Moreover, MPV was positively correlated with ambulatory diastolic blood pressure in essential hypertension. A very similar study demonstrated that MPV values were significantly higher in the prehypertensive group than in the control group (respectively, 10.41 +/- 0.93fl  vs.  9.56 +/- 1.04fl, p < 0.01) [22]. Additionally, MPV was positively correlated with the systolic blood pressure. The present study also displayed a significant increase in MPV in prehypertensive subjects and is therefore in agreement with the previous investigations that pointed out that MPV values increase gradually with the severity of hypertensive complications.

Furthermore, the Atherosclerosis Risk in Communities (ARIC) Study reported that patients with microaneurysms, retinal hemorrhages, and soft exudates have two to three times greater risk of developing clinical stroke over a period of three years than those with no retinal lesions, independent of elevated lipid levels, diabetes mellitus, hypertension, and other risk factors [23]. The distribution of patients with HR among different age groups revealed that the prevalence was maximum in older people, between 71 to 80 years of age, partially agreeing with other studies [24]. In the present study, however, the patients in age group 71-80 constituted only about 5.8% of the total sample size while in the aforementioned study it was 46.6%. This large difference between the present and previous studies may reflect the non-probability purposive sampling technique.

It should be noted that in the present and the previous studies not all patients with HR exhibited increased platelet activation [25-27]. Thus, other factors must also be involved in some cases. It might be relevant to note that the autoregulation of retinal circulation fails as blood pressure increases beyond a critical limit, and there are cases in which retinopathy was resolved despite the persistence of high blood pressure [26]. Nevertheless, the presence of high MPV values in HR and the correlation of the amount of MPV with the severity of HR imply that MPV may be involved in the mechanism of HR.

## Conclusions

Assessment of MPV is a cheap routine test for early monitoring of hypertensive patients and may help in predicting the risk of HR. As such, MPV testing could be used to decide whether preventative treatments should be used to prevent the severe complication of retinopathy. 
